# Kolmogorov–Arnold Networks for Sensor Data Processing: A Comprehensive Survey of Architectures, Applications, and Open Challenges

**DOI:** 10.3390/s26082515

**Published:** 2026-04-19

**Authors:** Antonio M. Martínez-Heredia, Andrés Ortiz

**Affiliations:** 1 Department of Communications Engineering, University of Malaga, 29071 Malaga, Spain; antoniomanuel.martinezheredia@uma.es; 2 Higher Polytechnic School, Nebrija University, 28015 Madrid, Spain

**Keywords:** Kolmogorov–Arnold Networks (KANs), sensor data processing, industrial sensing, medical imaging, remote sensing, interpretability, deep learning

## Abstract

Kolmogorov–Arnold Networks (KANs) have recently gained increasing attention as an alternative to conventional neural architectures, mainly because they replace fixed activation functions with learnable univariate mappings defined along network edges. This design not only increases modeling flexibility but also makes it easier to interpret how inputs are transformed within the network while maintaining parameter efficiency. KANs are particularly well suited for sensor-driven systems where transparency, robustness, and computational constraints are critical. This study provides a survey of KAN-based approaches for processing sensor data. A literature review conducted from 2024 to 2026 examined the deployment of KAN models in industrial and mechanical sensing, medical and biomedical sensing, and remote sensing and environmental monitoring, utilizing a Preferred Reporting Items for Systematic Reviews and Meta-Analyses (PRISMA)-based methodology. We first revisit the theoretical foundations of KANs and their main architectural variants, including spline-based, polynomial-based, monotonic, and hybrid formulations, to structure the discussion. From a practical standpoint, we then examine how KAN modules are integrated into modern deep learning pipelines, such as convolutional, recurrent, transformer-based, graph-based, and physics-informed architectures. KAN-based models demonstrate comparable predictive performance as conventional machine learning models, while having fewer parameters and more interpretable representations. Several limitations persist, including computational overhead, sensitivity to noisy signals, and resource-constrained device deployment challenges. Real-world sensor systems encounter significant challenges in adopting KAN-based models, including scalability in large-scale sensor networks, integration with hardware architectures, automated model development, resilience to out-of-distribution conditions, and the need for standardized evaluation metrics. Collectively, these observations provide a clearer understanding of the current and potential limitations of KAN-based models, offering practical guidance on the development of interpretable and efficient learning systems for future sensor equipment applications.

## 1. Introduction

Modern sensor systems play a central role in a wide range of cyber–physical and data-driven applications, including industrial monitoring, predictive maintenance, medical diagnosis, intelligent transportation, environmental observation, and smart grids. Extensive research has documented the capabilities of these systems [1,2], which can utilize large-scale sensor networks to gather high-dimensional real-time data streams on an ongoing basis thanks to developments in sensing technologies and increased access to edge computing infrastructure and connectivity. Noise, nonlinear responses, and uneven sampling across spatial and temporal scales frequently compromise sensor measurements, thereby complicating data analysis and decision-making in real-world sensing settings.

Deep learning has become a dominant paradigm for processing sensor data, achieving strong results in tasks such as time-series forecasting, hyperspectral image classification, medical image analysis, intrusion detection, and radar or Unmanned Aerial Vehicle (UAV)-based perception [3,4,5,6,7,8]. Deep learning models such as Convolutional Neural Networks (CNNs), Recurrent Neural Networks (RNNs), and Transformer-based models have demonstrated robust performance capabilities. Despite these advances, many deep learning models still operate as opaque “black boxes”, offering limited insight into how predictions are generated or which sensor features drive the decision process. This lack of interpretability raises concerns in safety-critical or regulation-sensitive domains such as healthcare, industrial monitoring, and critical infrastructure protection [9]. Furthermore, conventional Multi-Layer Perceptrons (MLPs) and convolutional architectures rely on fixed activation functions and dense weight matrices, which can limit parameter efficiency and scalability when modeling complex, high-dimensional sensor signals [10,11].

Kolmogorov–Arnold Networks (KANs) have recently gained increasing attention as an alternative to traditional perceptron-based architectures, mainly due to their interpretability and flexible function approximation capabilities [10,12,13]. Inspired by the Kolmogorov–Arnold Representation Theorem (KART) [14,15,16], KANs replace fixed scalar weights with learnable univariate functions defined on network edges, typically parameterized through basis expansions such as B-splines or polynomial families [17,18]. This functional representation enables more flexible modeling of nonlinear relationships while making learned transformations easier to visualize, which can improve interpretability and parameter efficiency [18,19,20]. Recent research has explored KAN-based models across multiple application domains [21] and has extended the original formulation to several architectural variants, including Chebyshev- and Jacobi-based KANs, monotone and input-convex formulations, and hybrid architectures that integrate KAN modules into CNNs, Long Short-Term Memory (LSTMs), Transformers, Graph Neural Networks (GNNs), and Physics-Informed Neural Networks (PINNs) [3,22,23,24,25,26,27,28,29].

In parallel with these architectural developments, the use of KAN-based models in sensor-driven scenarios has been investigated in an increasing number of studies. These applications span multiple domains, including industrial and mechanical sensing, structural health monitoring, medical imaging and biosignal analysis, hyperspectral and remote sensing, energy systems, transportation and mobility, and cyber–physical security [4,5,26,30,31,32,33,34,35,36,37,38,39,40,41,42,43]. Several studies report that KAN-based architectures can match or outperform state-of-the-art deep learning models while using fewer parameters, providing smoother function approximations, or producing interpretable functional representations [10,11,18,19,30]. These characteristics make KANs particularly attractive for sensor data processing, where predictive performance, model transparency, and computational efficiency are often equally important.

Despite the rapid growth of KAN research, the literature remains fragmented across application domains and architectural variants. Existing surveys primarily focus on theoretical foundations, approximation properties, or general developments of KAN architectures without providing a unified perspective on sensor-driven applications. Researchers and practitioners working with sensing systems may find it difficult to determine which KAN architectures are most suitable for specific sensor modalities, data characteristics, and operational constraints.

To clarify the novelty of this work with respect to previous reviews, Table 1 compares the scope of existing survey papers on KANs. Early reviews, such as the one by Dutta et al. [44], provided an initial mapping of the rapid expansion of KAN variants shortly after their introduction. More recently, Wang et al. [45] and other researchers [12,13] have offered valuable theoretical insights and broader research directions. Yamak et al. [46], for instance, focused specifically on time-series forecasting (TSF), with limited coverage of IoT-related applications. These studies are limited to specific tasks or are domain-agnostic, and none provide a systematic analysis of sensing modalities, hardware constraints, and cross-domain challenges essential for processing sensor data. The survey adopts a sensing-oriented approach, investigating KAN-based models across diverse fields, including biomedical sensing and remote sensing, and addressing the challenges posed by high-dimensional, noisy, and real-time sensor data.

Unlike previous surveys that primarily emphasize theoretical aspects or general architectural developments, this work systematically analyzes the deployment of KAN models across heterogeneous sensing modalities, including industrial monitoring, biomedical signals, and remote sensing pipelines.

The main contributions of this survey are summarized as follows:

- A sensor-centric perspective on KAN research. We review KAN-based models specifically in the context of sensor data processing, emphasizing domain-specific requirements and practical constraints rather than treating them as purely general-purpose architectures.
- A structured taxonomy of KAN applications in sensing. The literature is organized according to sensing domain, data modality, learning task, and architectural integration strategy, providing a clearer view of how KANs are used across different scenarios.
- A comparative analysis of hybrid KAN architectures. We examine how KAN modules are incorporated into convolutional, recurrent, Transformer-based, graph-based, and physics-informed models within sensing applications.
- A cross-domain synthesis of empirical trends. We analyze the reported results in terms of predictive performance, interpretability, parameter efficiency, and computational trade-offs across industrial, biomedical, and remote sensing tasks.
- An agenda for future research. We identify open challenges related to scalability, hardware efficiency, robustness to noisy sensor signals, out-of-distribution behavior, and the need for standardized benchmarking.

This survey intentionally focuses on sensing systems rather than general machine learning applications to analyze the specific challenges associated with noisy, high-dimensional, and real-time sensor data streams.

## 2. Background and Theoretical Foundations

Recent open-source implementations, such as PyKAN and related research prototypes, have simplified the process of experimenting with KANs and integrating them into contemporary deep learning frameworks. This section provides a summary of the theoretical underpinnings of these models and examines their applicability to sensor data.

### 2.1. Sensor Data Characteristics and Modeling Challenges

Sensor data play a central role in many cyber–physical systems, including industrial monitoring, healthcare, intelligent transportation, environmental observation, and energy management [1,2]. Advances in sensing technologies and communication infrastructures have enabled the deployment of large-scale sensor networks that continuously generate high-dimensional data streams.

Sensor data can take the form of multivariate time series, images and videos, hyperspectral cubes, radar or Light Detection and Ranging (LiDAR) signals, or more complex spatiotemporal representations, depending on the sensing modality used. These data often exhibit noise, missing values, nonlinear relationships, and strong correlations between channels or spatial locations, making reliable modeling and interpretation challenging. Consequently, machine learning models for sensor data processing must achieve not only high predictive accuracy but also robustness, computational efficiency, and interpretability under practical deployment constraints. The ability to model nonlinear calibration drifts or sensor-specific response patterns is particularly important [47,48], for instance, architectures that can adapt to the physical range of each input channel are required to capture a gas sensor’s saturation or a pressure transducer’s hysteresis.

### 2.2. From Perceptrons to Kolmogorov–Arnold Networks

Classical feedforward neural networks, such as MLPs, approximate nonlinear functions through affine transformation and fixed activation function compositions. This paradigm is based on the Universal Approximation Theorem (UAT), which states that a network with a single hidden layer and appropriate width, combined with a fixed, nonlinear activation function (such as ReLU or Sigmoid), can approximate any continuous function on a compact domain [49,50,51]. Unfortunately, the UAT does not specify a maximum network width, often resulting in unwieldy and over-parameterized models when aiming to capture the variability seen in sensor data. Furthermore, MLPs are typically treated as opaque “black boxes”, making it difficult to understand which sensor features influence the output.

The KART provides an alternative viewpoint on multivariate function approximation. It states that any continuous multivariate function can be represented as a finite superposition of continuous univariate functions combined through addition operations [14,15,16,52]. KANs build on this idea by replacing fixed scalar weights with learnable univariate functions defined along network edges. This functional representation enables more flexible modeling of nonlinear relationships while making learned transformations easier to interpret, improving interpretability and parameter efficiency.

### 2.3. Mathematical Foundations and Parameterization

To better understand the paradigm shift introduced by KANs, consider a continuous function $f:{\left[0,1\right]}^{d}\to R$. The KART establishes that there exist continuous univariate functions $\Phi_{q}$ and $\phi_{q,p}$ such that

(1) $$f\left(x_{1},\ldots ,x_{d}\right)=\sum_{q=1}^{2d+1}\Phi_{q}\left(\sum_{p=1}^{d}\phi_{q,p}\left(x_{p}\right)\right)$$

where $\phi_{q,p}:\left[0,1\right]\to R$ and $\Phi_{q}:R\to R$ are univariate functions. Although the original theorem was deemed impractical due to the potentially non-smooth (fractal) nature of these functions, modern KANs address this limitation by parameterizing the univariate components using basis expansions such as B-splines or polynomial families [10,17,18].

In modern implementations like PyKAN, the activation function ϕ(x) on a given edge is defined as a residual block to ensure numerical stability and a baseline response:

(2) $$\phi \left(x\right)=w_{b}b\left(x\right)+w_{s}s\left(x\right)$$

where b(x) denotes the basis function, is commonly activated by the Sigmoid Linear Unit (SiLU), s(x) represents the learnable spline function, and $w_{b}$ and $w_{s}$ are the weight parameters. This formulation allows the model to adapt the spline shape to the characteristics of the sensor signal while maintaining stable gradient propagation.

### 2.4. Spline-Based KAN Parameterization and Grid Extension

A common implementation of spline-based KANs relies on B-spline bases. Let ${\left\{t_{m}\right\}}_{m=1}^{M}$ denote a knot sequence and ${\left\{B_{k}\left(x\right)\right\}}_{k=1}^{K}$ be the associated B-spline basis functions of degree *p*. Each univariate edge function $g_{ij}$ is expressed as

(3) $$g_{ij}\left(x\right)=\sum_{k=1}^{K}\alpha_{ijk}\;B_{k}\left(x\right)$$

where the coefficients $\alpha_{ijk}$ are optimized jointly with the network parameters. This introduces a natural mechanism for regularization. Adjusting the number of knots (*K*), practitioners can control the smoothness of sensor-to-output mapping. In noisy environments, a coarser grid acts as a low-pass filter, reducing overfitting to high-frequency noise. Conversely, the “grid extension” property enables progressive refinement of the model without retraining it from scratch.

Furthermore, only the control points near that specific input range are updated during backpropagation when a localized anomaly or transient drift occurs in a sensor reading because B-splines possess local support. This localized learning mechanism helps mitigate the “catastrophic forgetting” typically observed in global MLP updates, making KANs highly suitable for continuous learning in dynamic cyber–physical systems.

### 2.5. Taxonomy of Basis Functions and Hybrid Variants

The flexibility of the KAN framework is primarily attributed to the variety of basis functions employed for edge-wise parameterization. Unlike traditional MLPs, KANs allow the adaptation of functional approximations to the specific characteristics of sensor data. Table 2 details the based on functions evaluated in this study, ranging from locally controlled splines to global orthogonal polynomials.

The KAN families listed in Table 2 have been illustrated in recent studies using B-splines [56], Chebyshev polynomials [57], Legendre polynomials [26], Hermite splines [24], and RBFs [58], showing their versatility across various sensing applications.

Based on these formulations, several variants of KAN have emerged to address different sensing modalities (see Table 3). Hybrid architectures are increasingly common, where KAN modules serve as drop-in replacements for MLP blocks within established backbones, such as CNNs for hyperspectral imaging or GNNs for distributed sensor networks.

### 2.6. Theoretical Advantages for Sensing Systems

The transition from MLPs to KANs in sensor data processing offers three primary advantages:

1. Interpretability: KANs can directly visualize the relationship between a specific sensor input (e.g., temperature) and the subsequent layer’s activation, offering a more transparent alternative to traditional weights.
2. Parameter efficiency: KANs often achieve comparable or superior accuracy to MLPs with significantly fewer parameters, which is critical for edge computing and low-power sensor nodes.
3. Scalable resolution: The grid extension property supports a gradual refinement of model resolution, enabling practitioners to increase complexity as needed without full retraining.

## 3. Literature Search Methodology

The review process was conducted following the Preferred Reporting Items for Systematic Reviews and Meta-Analyses (PRISMA) framework to improve transparency and reproducibility. A systematic literature review was carried out using two interdisciplinary scientific databases, Web of Science (WoS) and Scopus, selected for their comprehensive coverage of computer science, engineering, and sensing research areas.

The search strategy combined Boolean operators with terms related to both Kolmogorov–Arnold Networks and sensing applications. The primary query followed the structure: (“Kolmogorov–Arnold Networks” OR “KAN”) AND (“industrial” OR “mechanical” OR “sensor” OR “signal processing” OR “monitoring” OR “internet of things” OR “remote sensing” OR “diagnostics”). Whenever supported by the database interface, searches were limited to title, abstract, and keyword fields. The final search update was carried out in March 2026.

Additional articles indexed in IEEE Xplore were identified through backward reference screening and forward citation tracking to improve the coverage of applied engineering and machine learning studies. This complementary step allowed us to capture relevant studies that may not yet have been fully indexed in multidisciplinary databases.

The search window covered January 2024 to March 2026, which corresponds to the period following the introduction of Kolmogorov–Arnold Networks in the recent deep learning literature after the seminal work by Liu et al. [10]. Earlier publications were only considered to provide theoretical context related to the KART and were not included in the application-oriented review corpus.

### 3.1. Selection Criteria and Data Extraction

The records were screened in successive stages, including duplicate removal, title and abstract screening, and full-text eligibility assessment. Screening was performed manually by the authors. When the relevance of the study was uncertain, the final decision was made through discussion, considering the scope of the review and each study’s methodological completeness.

Studies were included when they met the following criteria:

- Inclusion criteria: (1) original research implementing KAN-based architectures applied to physical sensor data or sensor-derived signals; (2) studies reporting empirical evaluation on sensing-related datasets; (3) studies providing comparative results against baseline models such as MLPs, CNNs, RNNs, or Transformers; and (4) studies discussing relevant aspects such as interpretability, parameter efficiency, robustness, or edge-deployment feasibility.

The following exclusion criteria were applied:

- Exclusion criteria: (1) purely theoretical works without empirical validation on sensing datasets; (2) abstracts, posters, editorials, or articles lacking sufficient methodological detail; (3) duplicate records across databases; and (4) studies with insufficient experimental description or architectural detail to support a meaningful comparison.

Citation count was not used as a strict exclusion rule because the field is rapidly evolving. The qualitative assessment viewed it as a complementary indicator of publication maturity. Recent uncited studies were retained when they provided sufficient technical detail and clearly differentiated their methodological contributions.

The extracted information for each study included the sensing domain, data modality, learning task, KAN variant or hybrid architecture, baseline models, evaluation metrics, and reported benefits concerning interpretability, parameter efficiency, and deployment feasibility.

### 3.2. Search Results and Study Selection

The initial search yielded 862 records. After removing duplicates, 259 unique articles remained for title and abstract screening. During this stage, 104 records were excluded because they were outside the scope of the review or did not involve sensor-related applications. The remaining 155 articles were evaluated in full-text format. After eligibility assessment and technical quality screening, 97 studies were excluded, resulting in a final corpus of 58 articles for in-depth analysis.

Table 4 summarizes the number of retrieved and selected studies across the sensing domains considered in this review.

**Figure 1:**
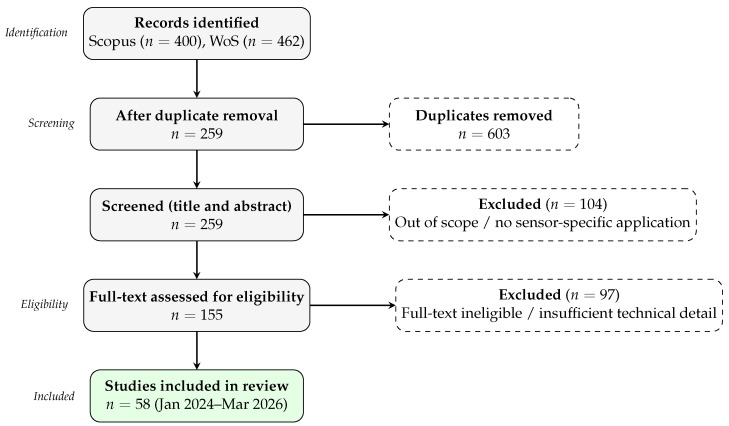
PRISMA-inspired flow diagram of the literature search and study selection process. Records were identified from Scopus and WoS for the period January 2024 to March 2026.

Figure 1 illustrates the PRISMA-inspired flow diagram that summarizes the study selection process.

Research on KAN-based sensing is increasingly moving from purely theoretical concepts to practical applications in industrial monitoring, biomedical analysis, and remote sensing.

**Figure 2:**
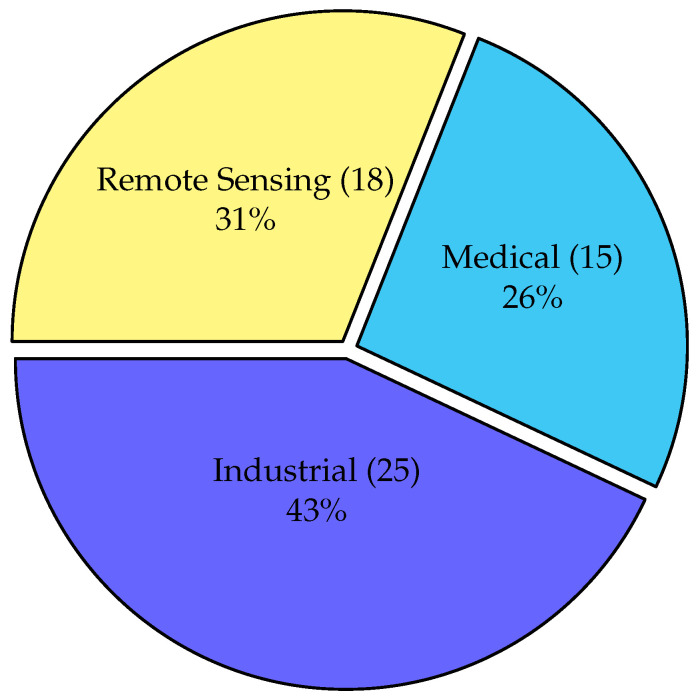
Distribution of reviewed KAN-based studies across the three main sensing domains (N=58).

As shown in Figure 2, the final corpus is relatively balanced across the three main sensing domains, with a slight predominance of industrial and mechanical sensing (43%). Remote sensing and environmental monitoring account for 31% of the studies, whereas medical and biomedical sensing represent 26%. This distribution suggests that KAN adoption is expanding across domains characterized by nonlinear data, heterogeneous modalities, and increasing demand for interpretable and efficient models.

### 3.3. Keyword Network and Research Landscape

**Figure 3:**
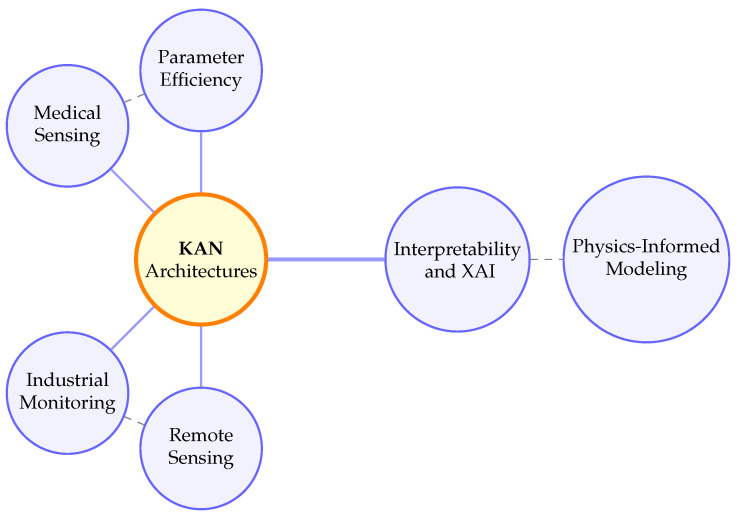
Conceptual map of KAN-based sensor research highlighting the relationships between sensing domains and cross-cutting research themes.

To provide a qualitative overview of the thematic relationships identified in the reviewed literature, a conceptual keyword network was constructed (Figure 3). The figure summarizes the main conceptual links observed across the reviewed studies and should be interpreted as a qualitative synthesis rather than a formal bibliometric co-occurrence analysis.

The diagram highlights the central role of KAN architectures in connecting domain-specific sensing applications with recurring cross-cutting research themes such as interpretability, parameter efficiency, and physics-informed modeling.

### 3.4. Classification of the Reviewed Literature

After the selection process, the final corpus of 58 studies was organized using two complementary classification perspectives: the sensing domain addressed and the type of KAN architecture employed.

From the application perspective, three main domains were identified: (i) industrial and mechanical sensing, (ii) medical and biomedical sensing, and (iii) remote sensing and environmental monitoring. Industrial sensing studies commonly address predictive maintenance, structural health monitoring, vibration analysis, and diagnostics of industrial processes. Medical sensing research focuses on medical imaging, physiological signal analysis, and clinical decision-support systems. Remote sensing applications include hyperspectral image classification, geospatial monitoring, environmental sensing, and Earth observation tasks.

The reviewed works were classified into spline-based KAN models, polynomial-based KAN variants including Chebyshev or Legendre expansions, and hybrid architectures that combine KAN modules with convolutional, transformer-based, recurrent, or graph-based learning frameworks. This dual classification provides a structured basis for the domain-specific analysis presented in the following sections.

### 3.5. Classification Taxonomy

**Figure 4:**
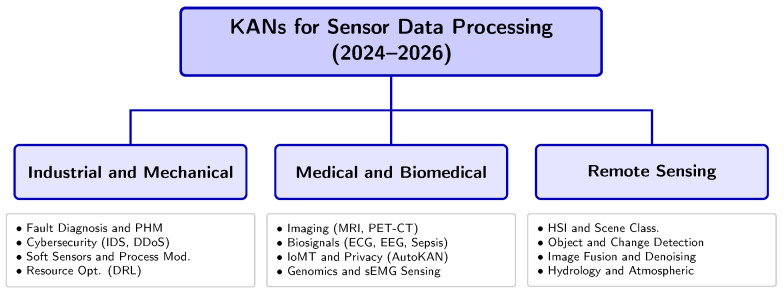
Classification taxonomy of KAN-based architectures in the sensor domain, organized by application field and specific analytical task.

We developed a domain-driven taxonomy to structure the analysis of the selected literature, which categorizes KAN-based architectures by sensing modality and operational environment (see Figure 4). This classification reflects the transition of KAN-based models from theoretical constructs to more specialized solutions across different sensing fields.

## 4. Industrial and Mechanical Sensing

### 4.1. Fault Diagnosis and Industrial Monitoring

KAN-based models, including tool wear state prediction and bearing fault diagnosis, have been used to monitor the condition of industrial machinery using sensor data. They have also been incorporated into real-time anomaly detection frameworks for Industrial Internet of Things (IIoT) environments, such as water treatment plants and automated production lines. A significant portion of the selected literature focuses on fault diagnosis in rotating machinery.

Rigas et al. [30] proposed a framework for bearing failure diagnosis, which achieved perfect F1-scores, demonstrating that KAN architectures can provide interpretable symbolic representations that enable engineers to understand the impact of specific signal components. Yan et al. [59] also developed a CNN-1D-KAN model for cross-domain fault diagnosis, demonstrating that KAN-linear layers improve the model’s ability to generalize across different load conditions compared to standard MLPs.

Data efficiency in industrial environments has also been investigated. Luna-Villagomez and Mahalec [60] analyzed the Tennessee Eastman Process benchmark and demonstrated that KAN autoencoders (KAN-AE) require significantly fewer samples (2500 versus 30,000) than traditional orthogonal autoencoders to achieve comparable detection performance. Zhang et al. [61] and Luo et al. [62] showed the benefits of KAN-based models in spectral analysis for high-precision inspection tasks, such as Laser-Induced Breakdown Spectroscopy (LIBS) and Prompt Gamma Neutron Activation Analysis (PGNAA), achieving $R^{2}$ values over 0.95 in elemental quantification.

KAN architectures have also been used to optimize complex industrial processes. Ma et al. [63] integrated KANs with genetic algorithms (GA) for laser welding optimization, while Ansar and Ashraf [33] proposed a modified KAN loss function incorporating the Pearson correlation coefficient (PCC) to improve thermal efficiency in 660 MW power plants.

Beyond traditional industrial assets, monitoring applications have expanded to include biological systems. Wang et al. [64] introduced the lightweight HRNet with Dim-Channel and Space Gate Attention Using Kolmogorov–Arnold Networks (HRDS) model, which reduces the number of parameters by 73% for animal keypoint detection, enabling real-time health monitoring in resource-constrained environments. Additional studies include tool wear recognition using Vision Transformer–KAN hybrids [65], steel plate fault detection with Set Transformer–KAN models [66], and multi-step time-series forecasting using the Convolutional KAN (C-KAN) architecture [3].

Huang et al. [67] used KANs for symbolic regression (SR) to construct gray-box Simulation Program with Integrated Circuit Emphasis (SPICE) models that outperform traditional Artificial Neural Network (ANN) “black-box” approaches. By generating explicit mathematical formulas, the method ensures high interpretability and accelerates the Design Technology Co-Optimization (DTCO) process. Simulations of an 11-stage ring oscillator at the 12 nm node validate that these expressions accurately capture current-voltage (I-V) characteristics, providing a streamlined, high-precision solution for advanced semiconductor modeling.

Recent architectures have also targeted the non-stationary nature of sensor data. Hybrid Imaginary Exponential KAN (HiKAN) [68] specifically addresses this setting. Using an Imaginary Exponential basis (IEKAN), this model achieves a 10.3% improvement in MSE over traditional methods, providing the symbolic transparency necessary for high-stakes SCADA environments.

### 4.2. Industrial IoT (IIoT) and Cybersecurity

The security of cyber–physical systems (CPSs) and IIoT infrastructure represents another critical application area for KAN models. In this context, KAN-based architectures have been proposed as lightweight alternatives for Intrusion Detection Systems (IDSs).

Ghorbani et al. [69] and Chen et al. [70] developed KAN-based frameworks for detecting Distributed Denial-of-Service (DDoS) attacks and network intrusions, reporting high detection accuracy with reduced computational overhead. For resource management in IIoT environments, Wu et al. [71] incorporated KAN modules into a reinforcement learning framework to optimize computation offloading strategies. Abudurexiti et al. [72] proposed an unsupervised anomaly detection architecture, combining a Time Convolutional Network (TCN), a Variational AutoEncoder (VAE), and KAN components, to enable the explainable detection of irregular patterns in sensor streams.

### 4.3. Soft Sensors and Process Modeling

Additionally, KAN modules have been integrated into graph-based and temporal neural architectures that can capture complex dynamic relationships in industrial processes.

Representative examples include the adaptive Kolmogorov–Arnold-based Graph Neural Network (AKGNN) proposed by Yang et al. [73], which addresses cases where the underlying graph structure of the process is unknown. Sun et al. [32] introduced a multi-timescale sensing architecture that combines TCN, Bidirectional Long Short-Term Memory (BiLSTM), and KAN components to capture temporal dependencies at different scales. Similarly, Yao et al. [74] developed the Kolmogorov–Arnold graph convolutional aggregation temporal convolutional network model (KAGCN-KATCN) for quality prediction in chemical production units. In environmental process monitoring, Cheng et al. [75] applied KAN architectures to wastewater treatment systems, demonstrating their ability to model complex bio-electrocatalytic interactions relevant to energy recovery processes. A similar emphasis appears in the work of Sánchez-Gendriz et al. [76], who used KANs to extract interpretable symbolic expressions for Water Quality Indices (WQIs). Their framework achieved $R^{2}>0.96$ and outperformed traditional ANN baselines even after sensor pruning, effectively accelerating DTCO workflows. Similarly, Fricz et al. [77] evaluated KANs in aniline synthesis, replacing black-box models with explicit formulas. KAN-derived expressions can achieve a high level of complexity, necessitating expert interpretation via Shapley values, yet they provide a viable means to optimize industrial costs and product quality.

### 4.4. Prognostics and Health Management (PHM)

KAN-based hybrid architectures have been investigated for PHM, particularly for estimating Remaining Useful Life (RUL) and predicting component failures.

He et al. [78] integrated a Transformer–KAN architecture with a nonlinear Wiener Process (WP) model to analyze the degradation of rotating machinery, where the KAN component acts as the drift function. Xiao and Wang [79] also developed a Performer–KAN model for failure prediction in Insulated-Gate Bipolar Transistors (IGBTs), providing a scalable framework for real-time health monitoring in high-reliability power electronics systems.

To provide a structured overview of the reviewed approaches, Table 5 summarizes representative KAN-based architectures, their application domains, and their key contributions in industrial and mechanical sensing. Although numerical results are omitted for brevity, the table offers a qualitative comparison of architectural trends and clarifies the roles played by KANs across different sensing settings.

Despite these promising results, several limitations have been identified across industrial sensing studies. KAN-based models exhibit increased sensitivity to the high-frequency noise inherent in vibration and acoustic emission signals. Without adequate preprocessing, the expressive power of high-order splines may lead to overfitting to noise artifacts rather than capturing meaningful fault signatures. Most evaluated benchmarks are small to medium in scale, and the stability of KAN training on large-scale Supervisory Control and Data Acquisition (SCADA) streams with thousands of simultaneous sensor channels has not yet been systematically validated. The computational overhead of spline evaluation is a practical barrier to real-time deployment in high-frequency monitoring scenarios, where inference latency requirements typically range from milliseconds.

## 5. Medical and Biomedical Sensing

KAN-based architectures have been widely explored in medical sensing tasks involving both imaging and physiological time-series data. In medical imaging, typical inputs include Positron Emission Tomography–Computed Tomography (PET-CT) or Magnetic Resonance Imaging (MRI) scans, histopathology slides, and microscopic cell images, where the goal is to classify disease states, segment lesions, or detect subtle structural abnormalities [4,8,80]. These datasets are often high-dimensional, exhibit complex texture and intensity patterns, and may suffer from class imbalance or limited labeled samples. The constraints require the use of architectures that combine robust representational capacity with interpretability.

In this context, several studies integrate KAN modules into convolutional and Transformer-based backbones. For instance, Sait et al. [80] developed a hybrid model for lung cancer classification using PET-CT images, combining MobileNet V3 and LeViT with KAN layers to achieve an accuracy of 99.0%. Similarly, in respiratory health monitoring, Chau et al. [42] used KAN-based architectures to classify Quantitative Computed Tomography (QCT) data, reaching an accuracy of 97.35%.

To further address the nonlinear characteristics of medical images, the Multi-Scale Feature Kolmogorov–Arnold Network (MSFKAN) model [81] introduces a multiscale feature joint prediction network. The MSFKAN model achieves 97.48% accuracy in brain tumor (MRI) classification by concatenating convolutional blocks to produce features at different scales and applying a spatial attention mechanism, which is then weighted by a KAN layer to demonstrate improved robustness against adversarial perturbations.

A notable development in medical image segmentation and classification is the introduction of hybrid encoder architectures such as Kolmogorov–Arnold Convolutional Network (KACNet) [82], U-KAN [83], and KANSeg [84]. KACNet introduces the KANConv module, which integrates KAN layers into convolutional networks to address the edge-blurring limitations associated with fixed activation functions. A multistage architecture enables the combination of local features through CNN layers and global information via KAN layers with B-spline functions, resulting in state-of-the-art performance on various 2D segmentation and 3D classification benchmarks. KANSeg uses a KAN-Activated Convolution module (KAN-ACM) and a KAN bottleneck module (KAN-BM) to represent nonlinear anatomical features and enhance segmentation performance in regions with indistinct organ boundaries, achieving Dice scores of up to 90.99% on the Automated Cardiac Diagnosis Challenge (ACDC) dataset.

In clinical pathology, KAN-based architectures have also benefited diagnostic efficiency. The K2AN and KAN-C-Norm models [85] improve the recognition of colony-forming units (CFUs) in urinary tract infections (UTI) by 7.83% compared with MLP-based benchmarks. Additional architectures include Deep equilibrium Kolmogorov–Arnold Networks (DEQ-KANs) [86] for classification in unbalanced datasets, Group-Dynamic KANSformer (GDKansformer) [40], which integrates group-dynamic KAN modules with Vision Transformers, and Sparse-Causal KANSformer (SCKansformer) [41], which targets bone marrow cell recognition.

From a mathematical perspective, a generic medical image classification task can be formulated as learning a mapping $F_{\theta }:R^{H\times W\times C}\to \left\{1,\ldots ,C_{\mathrm{cls}}\right\}$. A typical backbone computes a feature map $u=\Phi_{\theta_{1}}\left(x\right)\in R^{D}$, which is then mapped to class logits using a KAN head:

(4) $$z_{i}=\sum_{j=1}^{D}g_{ij}\left(u_{j}\right)+b_{i},\;i=1,\ldots ,C_{\mathrm{cls}},$$

where the univariate functions $g_{ij}$ are parameterized by spline or polynomial bases.

Beyond imaging tasks, KAN architectures have also been applied to physiological time-series data generated by medical sensors. Applications include electroencephalogram (EEG) analysis for seizure detection, electrocardiogram (ECG) processing for abnormality recognition, and photoplethysmogram (PPG) analysis for monitoring heart rate and oxygen saturation monitoring. Hybrid models have shown more promising behavior, even though purely KAN-based models do not always match the performance of specialized recurrent architectures as evidenced by Hasan et al.’s [38] findings of an 80.15% accuracy compared to 95.37% for an LSTM in seizure prediction.

For example, the KAN-EEG model [39] demonstrated improved generalizability across datasets with diverse geographical distributions. Similarly, the Time-Constant Kolmogorov–Arnold Network (TCKAN) [87] was proposed for predicting mortality in patients with sepsis. The TCKAN model achieved Area Under the Curve (AUC) scores of up to 88.07% on the MIMIC-III and MIMIC-IV datasets by combining temporal sequences, constant clinical variables, and categorical International Classification of Diseases (ICD) codes.

Recent research has also extended KAN architectures to the Internet of Medical Things (IoMT) and privacy-preserving healthcare monitoring. The AutoKAN framework [88] introduces a lightweight federated anomaly detection architecture for monitoring patients with diabetes. By replacing traditional MLP layers with KAN layers within an autoencoder structure and employing adaptive thresholds, AutoKAN achieves over 99.5% accuracy while reducing the number of parameters by approximately 50%. This design facilitates real-time deployment on resource-constrained edge devices while preserving patient privacy through federated learning.

Beyond imaging and biosignals, KANs have also demonstrated potential in biochemical sensing and genomics. Yadalam et al. [89] applied KAN architectures to predict drug–gene associations for HDAC1 inhibitors, achieving an accuracy of 96.49%. Similarly, the TCNN-KAN model [90] for gesture recognition using surface Electromyography (sEMG) signals achieved 98.38% accuracy while employing unstructured pruning to enable real-time edge-sensing deployment.

To provide a structured overview of the reviewed approaches, Table 6 summarizes representative KAN-based architectures, their application domains, and their key contributions in medical sensing. A direct numerical comparison is not always meaningful, given the diversity of modalities—ranging from biosignals (EEG and sEMG) to volumetric imaging (PET-CT) and genomics. Instead, the table offers a qualitative synthesis of how KAN-based components are used to enhance interpretability and feature extraction in clinical settings.

However, not all biomedical applications have yielded favorable results for KAN-based models. Pure KAN architectures have struggled to match the performance of Recurrent Neural Networks optimized for temporal dependencies in sequential biosignal processing. Hasan et al. [38] found that a standalone KAN achieved 80.15% accuracy in seizure prediction, compared to 95.37% for an LSTM baseline, suggesting that KANs may be insufficient for tasks requiring long-range temporal context. Additionally, the sensitivity of B-spline functions to noisy EEG and ECG signals often requires more robust preprocessing pipelines than those used with MLP-based models. The scalability of KAN models to large clinical cohorts—such as multi-center datasets beyond MIMIC-III and MIMIC-IV—also remains an open challenge that must be addressed before considering widespread clinical adoption.

## 6. Remote Sensing and Environmental Monitoring

The remote sensing community has rapidly adopted KANs to address the inherent nonlinearities of satellite and aerial data. Sensors in this domain, such as hyperspectral imaging (HSI) and synthetic aperture radar (SAR), capture complex physical interactions that traditional MLPs often fail to efficiently model. KANs have shown strong potential in signal processing for environmental sensing contexts and handling highly correlated, multidimensional data from hyperspectral imaging sensors. These architectures provide a more expressive and parameter-efficient alternative for high-dimensional Earth observation tasks, achieved by substituting fixed activation functions with learnable splines or polynomial bases.

### 6.1. Hyperspectral and Scene Classification

Initial efforts focused on land-cover and scene classification. Cheon et al. [91] introduced KonvNeXt, a KAN-RS model combining KAN layers with a ConvNeXt backbone, achieving 98.1% accuracy. Their study demonstrated that KANs consistently outperform MLPs in land-use and land-cover (LULC) tasks, offering superior interpretability via occlusion sensitivity.

For spectral–spatial feature extraction, Jamali et al. [92] proposed a hybrid 1D/2D/3D KAN architecture to improve feature learning. Roy et al. [93] further refined this concept using a spectral–spatial KAN specifically designed for HSI. Architectural innovations include HyperKAN [5], which modularly replaces linear layers with KANs, and HyperFKAN [94], which accelerates training by substituting B-splines with Fourier series. To address resource-constrained platforms, Qian et al. [95] introduced a hierarchical progressive fusion network (HPFN) using Fast KANs. Additionally, Han et al. [4] developed an enhanced fusion Transformer that integrates KAN encoders for superior HSI classification.

### 6.2. Object Detection, Segmentation, and Change Detection

The challenge of detecting small targets in UAV imagery led to the development of KSCNet [7]. This model incorporates KAN layers into a backbone alongside State Space Models (SSMs) to enhance global feature aggregation. For semantic segmentation under label scarcity, Zhang et al. [96] proposed SS-KAN, a self-supervised framework that is effective even with only 1% labeled data. In disaster monitoring, Wang et al. [97] introduced FloodKAN for SAR-based flood extraction.

In change detection, Liu et al. [34] developed the AutoEncoder Kolmogorov–Arnold Network (AEKAN), an unsupervised Siamese KAN autoencoder for multimodal scenarios. Similarly, Seydi et al. [98] demonstrated that a Chebyshev-KAN architecture using Chebyshev polynomials outperforms complex Transformers in hyperspectral change detection by effectively mitigating seasonal noise.

### 6.3. Image Fusion, Reconstruction, and Denoising

KANs have been applied to the continuous modeling and denoising tasks. Zhu et al. [99] developed Spatial–Frequency Sampling Implicit Neural Representation (SFIGNet) for HSI and Multispectral Imaging (MSI) fusion using implicit neural representations. Li et al. [100] integrated KANs with diffusion models in the KanDiff framework. Recently, super-resolution autoencoder-based KAN architectures have been explored to preserve high-frequency details and reconstruct fine spatial features. To address signal degradation, Ren et al. [101] developed a wavelet-based KAN for remote sensing image denoising, which separates noise and signal in the frequency domain, thereby preserving fine textures.

### 6.4. Environmental and Atmospheric Sensing

Beyond imaging, KANs have demonstrated high efficacy in hydrological and agricultural monitoring. Isik et al. [37] applied KANs for interpretable crop yield prediction in the U.S. Corn Belt, outperforming traditional regression models. For water quality monitoring, Saravani et al. [102] used KANs to predict Chlorophyll-a concentrations in global lake systems.

In atmospheric retrieval, Tao et al. [103] introduced a KAN–Transformer hybrid for Coherent Doppler Wind Lidar (CDWL), while Wang et al. [35] developed SwinKAN for radar-based weather extrapolation. Finally, Wu et al. [104] proposed KACNet for detecting hyperspectral anomalies.

To provide a structured overview of the reviewed approaches, Table 7 summarizes representative KAN-based architectures, their application domains, and their key innovations in remote sensing. These studies cover highly diverse tasks, including hyperspectral classification, UAV detection, image fusion, and weather forecasting. Their performance metrics are not directly comparable. Therefore, the table emphasizes the qualitative evolution of the architectures and the specific advantages that KANs offer when handling the nonlinearities inherent in satellite and aerial data.

## 7. Cross-Domain Discussion and Research Challenges

Whereas the previous sections focused on summarizing KAN architectures and applications across different sensing domains, this section offers a more critical synthesis of the reviewed literature. Instead of listing isolated results, we examine recurring cross-study trends, identify common limitations, and assess the practical implications of KAN-based models for real-world sensor data processing. The trade-offs between interpretability, computational efficiency, and deployability in realistic environments, such as edge and resource-constrained systems, are being prioritized, along with methodological issues that complicate standardized comparisons with conventional deep learning methods.

A systematic literature review reveals that KANs have transitioned from a theoretical novelty to a versatile tool in the pipeline of sensor data processing. This section synthesizes findings across various domains and identifies the core challenges that will shape future research. The reported performance improvements typically range from 1% to 6% in predictive accuracy compared with conventional MLP-based architectures, while achieving significant reductions in parameter count between 30% and 70%. The gains are primarily attributed to the high expressivity of the learnable edge-based activation functions, allowing KANs to approximate intricate sensor patterns with fewer neurons.

The majority of the reviewed studies were based on publicly available benchmarks, including MIMIC-III/IV for medical sensing, hyperspectral remote sensing datasets, and industrial vibration datasets commonly used in predictive maintenance research.

Simultaneously, the absence of standardized benchmarking protocols across sensing domains limits the direct quantitative comparison between studies.

The lack of consistent evaluation settings further complicates the direct comparison between KAN-based models and conventional deep learning approaches. The reported performance gains should be viewed with caution, as they may be partly due to dataset-specific characteristics or experimental conditions rather than the inherent benefits of the architecture.

Overall, while KAN-based architectures offer clear advantages in terms of interpretability and functional expressiveness, their computational overhead and lack of standardized evaluation protocols limit their practical adoption in sensor systems. In contrast to conventional deep learning models like CNNs and Transformers, which can leverage established optimization pipelines and standardized benchmarking frameworks, KAN models need further validation in carefully controlled and reproducible experimental settings. This underscores the inherent trade-off between interpretability and deployment efficiency, suggesting that hybrid architectures may offer a viable approach for reconciling these conflicting demands in practical sensing applications.

### 7.1. Structural Integration and Performance Trade-Offs

To provide a broader synthesis of the current KAN landscape, Table 8 summarizes how KAN layers have been integrated into established deep learning architectures for sensor-driven data. Beyond simple categorization, the table highlights the practical trade-offs associated with these hybrid designs.

For example, integrating KANs into Autoencoders (AE) improves the representation of nonlinear noise during sensor reconstruction, but it also introduces greater training complexity than standard MLP-based alternatives. Similarly, CNN-based integrations (e.g., C-KAN, SwinKAN) provide stronger spatial interpretability through activation visualization. However, the computational cost of spline evaluation remains a significant bottleneck, increasing inference latency on resource-constrained edge devices. Transformer-based integrations may reduce parameter reliance in attention mechanisms through KAN layers, but the lack of standardized benchmarking has long hindered the validation of their stability in large-scale SCADA deployments. Recent advances, such as HiKAN [68], have begun to address some of these structural limitations by improving KAN performance on high-dimensional industrial sequences. Nevertheless, training overhead and real-time viability remain central open challenges as discussed in Section 8.

### 7.2. Emerging Architectural Patterns in Sensing

A cross-domain analysis identifies three dominant integration strategies for KANs that move beyond the original “pure KAN” proposal to address the complexities of real-world sensor streams:

1. Hybrid Backbone–Head Architectures: This is the most prevalent pattern observed in approximately 65% of the reviewed studies. Researchers use a CNN or Transformer backbone for high-dimensional feature extraction and replace the final MLP classification/regression head with a KAN layer. This strategy, implemented in models such as KACNet and HyperKAN, leverages the representation power of KANs to model the final decision boundary more accurately than a simple linear layer.
2. Deep Functional Integration (Drop-in Replacements): KAN modules are embedded deeper in the network in U-KAN or SwinKAN architectures, replacing specific linear blocks within the encoder or decoder stages. This allows the network to learn nonlinear transformations at multiple abstraction scales, which is particularly effective for edge-sensitive tasks such as medical image segmentation and radar-based weather extrapolation.
3. Physics-Informed and Constrained Topologies: KANs enforce physical laws in domains such as structural health monitoring and energy systems. Imposing mathematical constraints, such as monotonicity for sensor calibration, convexity for material hyperelasticity, or periodicity for seasonal energy cycles, is significantly easier than imposing standard MLPs on univariate functions that are learnable curves rather than fixed weights.

### 7.3. Benchmark-Based Critical Analysis of Hybrid Spectral–Spatial Architectures

Recent hybrid remote sensing models combine complementary paradigms to capture spectral and spatial dependencies. A representative example is HSS-KAMNet [93], which integrates KANs, state space models, and attention mechanisms within a unified architecture.

The model is evaluated on three standard RGB datasets (EuroSAT, UCM, and AID) from a benchmarking perspective, addressing the lack of consistent multi-dataset validation highlighted in recent surveys, and as shown in Table 9, it outperforms baselines in terms of F1-score.

These benchmarks are restricted to RGB imagery and do not fully account for the complexities of HSI classification, where spectral dimensionality and inter-class similarity are significantly greater. In contrast, the study in [92] evaluates hybrid KAN-based architectures on three UAV-based hyperspectral datasets (QUH-Tangdaowan, QUH-Qingyun, and QUH-Pingan) using domain-specific metrics, such as overall accuracy (OA), average accuracy (AA), and Kappa coefficient (κ). Table 10 summarizes the benchmark comparison on hyperspectral datasets.

The HyperKAN benchmark [5] provides comprehensive evaluation of KAN-based architectures on multiple widely used hyperspectral datasets, including Indian Pines, Pavia University, Salinas, Houston, and KSC. In contrast to [92], which focuses on specific acquisition scenarios, this study adopts a broader multi-dataset evaluation protocol and demonstrates consistent improvements of KAN-based models over traditional architectures across different spectral–spatial learning paradigms.

Note that hyperspectral image classification studies typically report OA, AA, and κ, whereas RGB-based benchmarks rely on F1-score and AUC. Therefore, a direct metric-level comparison across these domains is not feasible, and the results must be interpreted within their respective evaluation protocols.

From a critical perspective, several limitations remain. First, the extremely high accuracy reported by EuroSAT (>99%) may indicate dataset bias and limited real-world generalization. The reported performance gains are unreliable due to the lack of statistical significance analysis. Third, although hyperspectral benchmarks introduce higher complexity, they remain geographically constrained and may not fully represent global variability.

Finally, the architectural complexity of hybrid models introduces additional computational overhead. In particular, KAN-based components rely on spline-based functional representations, which increase the inference latency compared to standard linear layers.

Hybrid spectral–spatial models achieve state-of-the-art performance across both RGB and hyperspectral datasets, but a significant limitation is the absence of standardized evaluation protocols and real-world validation.

### 7.4. Interpretability: Moving Toward a “Gray-Box” Paradigm

A recurring conclusion across the medical, industrial, and environmental sectors is that KANs move sensing models toward a “gray-box” paradigm. Although more complex than simple linear regression, the ability to visualize the learned univariate functions allows for a level of transparency that is often unattainable in traditional deep learning:

- Clinical and Expert Validation: Clinicians can visualize how a KAN-EEG model weighs specific frequency bands in real-time. This ensures that the model’s logic aligns with known physiological patterns rather than relying on abstract, uninterpretable weight matrices. In materials science and industrial process modeling, KANs mark a shift in symbolic regression [105], moving away from traditional genetic programming towards deep learning-driven model discovery. By directly learning from concise and interpretable mathematical data expressions, KANs can serve as a bridge between data-driven models and classical physics.
- Gray-Box Surrogate Modeling: KANs facilitate the extraction of simplified formulas that describe complex physical properties. This makes it possible to develop models that preserve the deep learning’s predictive strength while retaining formula-based analytical solutions’ transparency.
- Local Error Analysis: The shape of the B-splines can indicate input space regions where the sensor data are less reliable or where the model lacks sufficient training samples, providing an implicit uncertainty quantification.

### 7.5. The Parameter Efficiency vs. Compute Trade-Off

One of the most touted benefits of KANs is their parameter efficiency. Studies such as AutoKAN and HRDS have demonstrated that KANs achieve similar or superior accuracy to MLPs with a 50% to 70% reduction in parameter count. However, an important technical caveat emerges: parameter efficiency does not necessarily translate into computational speed.

**Figure 5:**
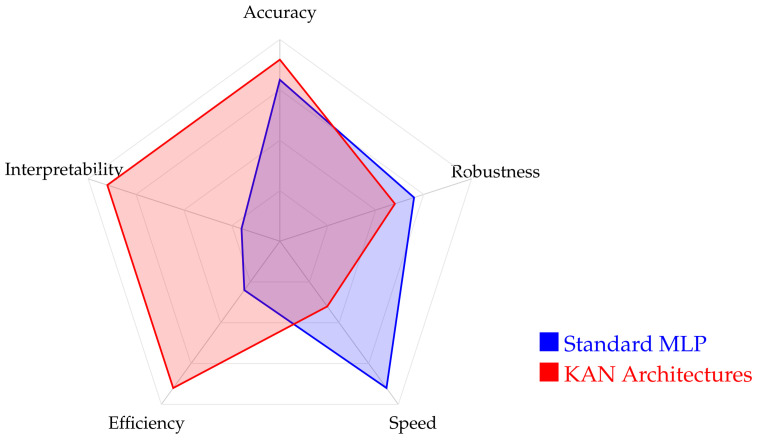
Comparative performance profile of KANs versus traditional MLPs across five key sensing metrics.

The qualitative performance trade-offs between KANs and traditional MLPs are visualized in the radar profile shown in Figure 5. Our comparative analysis shows that standard MLPs retain a significant advantage in computational speed due to well-established hardware optimization and lower architectural complexity per layer, but KAN architectures set a new benchmark for interpretability and parameter efficiency in sensor data processing. KANs’ ability to represent complex functional mappings with a significantly lower memory footprint, as evidenced by their high efficiency score, makes them an ideal candidate for resource-constrained edge devices. These findings suggest that KANs are a strategic choice for sensing applications where model transparency and energy efficiency are prioritized over raw inference throughput.

Nevertheless, these findings should be carefully interpreted because most of the reported gains were obtained under heterogeneous experimental conditions.

Traditional MLPs are heavily optimized for modern hardware (GPUs/TPUs) because their core operation is the General Matrix Multiply (GEMM), which is compute-bound and highly parallelizable. In contrast, KANs shift the bottleneck from being compute-bound to memory-bound:

- Gridvevaluation overhead: Evaluating a spline requires hardware to determine the grid interval at which a sensor reading falls and fetch corresponding control points. This introduces irregular memory access patterns that can degrade cache coherence.
- Training instability: KANs demand fewer parameters, yet their loss landscape can be more intricate due to the local support of B-splines, necessitating more advanced optimization strategies and longer training epochs than the rapid convergence of ReLU-based MLPs.

### 7.6. Noise Sensitivity and Preprocessing Requirements

While KANs excel at capturing nonlinear patterns, they are significantly more sensitive to signal quality than their MLP counterparts. The high expressive power of B-splines or high-order polynomials can lead to the “wiggling” effect, where the network fits high-frequency noise artifacts instead of the underlying signal.

This survey identifies a clear trend: KAN-based pipelines require more rigorous denoising (e.g., Wavelet transforms or Savitzky–Golay filters) before the data reaches the KAN layers. This adds a preprocessing overhead that must be considered when designing real-time sensing systems for noisy industrial or clinical environments.

This issue highlights an important limitation of KAN-based models compared with more robust deep learning architectures under noisy real-world sensing conditions.

### 7.7. Critical Research Challenges and Future Directions

Despite promising results, several gaps must be addressed to mature KAN technology for widespread industrial adoption:

- Standardization of basis selection: There is currently no consensus on which basis (B-splines, Chebyshev, Jacobi, or Fourier) best suits specific sensor modalities. Future research should focus on developing an “Auto-Basis” selection mechanism based on the spectral characteristics of the input data.
- Scalability and the curse of dimensionality: KANs suffer from a specific form of scaling. While the KART guarantees approximation, the number of univariate functions scales linearly with the number of input dimensions (2d+1). This can lead to an explosion in the number of edges for high-dimensional sensors (e.g., 500-channel hyperspectral cubes), necessitating sparse KAN topologies.
- Hardware-aware architectures: Specialized KAN kernels that employ standard matrix operations, such as those in Fast-KAN, or the investigation of neuromorphic and FPGA-based implementations are crucial for bridging the hardware–software gap and achieving low-latency edge sensing.

Taken together, these limitations indicate that KANs are not yet a fully mature alternative to conventional architectures in large-scale sensor deployments despite the encouraging results reported so far.

To provide a cross-domain perspective, Table 11 summarizes the main application domains, data modalities, representative KAN-based architectures, and their reported impact across industrial, medical, and remote sensing settings.

### 7.8. Limitations of This Review

Although this survey followed the PRISMA guidelines, several limitations must be acknowledged. First, the primary database search was restricted to Scopus and WoS; while IEEE Xplore was consulted during manual screening, some conference proceedings might still be excluded. Second, the search window (January 2024–March 2026) reflects the modern KAN era but excludes earlier foundational functional networks. Third, the “impact filter” (one minimum citation) managed the volume of preprints but may have excluded very recent, high-quality technical reports. Finally, the corpus is subject to publication bias because in early-stage KAN experiments, favorable results are more likely to be reported than neutral or negative outcomes.

## 8. Open Challenges and Future Directions

Despite the rapid progress and promising results of KANs in sensor-centric applications, several challenges remain before KAN-based models can be widely adopted in real-world sensing systems. These challenges span the methodological, computational, and practical aspects of large-scale sensor networks, including scalability, standardized benchmarks, and clearer design guidelines. The following subsections discuss the research directions that are likely to shape the future development of KAN models for sensor data processing.

### 8.1. Scalability and Large-Scale Sensor Networks

Most existing studies have evaluated KAN-based models on moderate-sized datasets or relatively small sensor configurations. However, modern cyber–physical systems and IoT infrastructures may involve thousands of sensors generating high-frequency data streams [1,27]. Scaling KAN architectures to such environments raises challenges related to training stability, memory consumption, and inference latency, particularly when using high-order spline bases or complex hybrid architectures [5,26].

Future studies should investigate scalable KAN variants and training strategies that can handle distributed sensor data. Promising directions include sparsity-inducing regularization, adaptive grid refinement, and distributed or federated learning frameworks. Notably, AutoKAN [88] represents a significant step forward by demonstrating federated anomaly detection in IoMT, ensuring privacy while reducing parameter complexity. Integrating KAN modules into graph-based learning frameworks also appears promising for modeling complex sensor network topologies [58,106].

### 8.2. Efficient Architectures and Training Strategies

Although KANs often provide higher expressivity per parameter, some implementations introduce computational overhead due to spline evaluations. The design of efficient KAN architectures that preserve interpretability while reducing memory requirements remains a priority.

Potential solutions include model pruning, adaptive basis selection, and hardware-aware optimization. Hybrid architectures in which KAN modules replace only selected components (such as MLP blocks) within larger networks, as seen in the KANConv module of KACNet [82], offer a viable path. Such designs, including the KAN-ACM and KAN-BM modules in KANSeg [84], achieve favorable trade-offs by combining the local feature extraction capability of CNNs with the global nonlinear mapping properties of KANs.

### 8.3. Out-of-Distribution Handling and Dynamic Grid Extension

A fundamental limitation of spline-based KANs in real-world sensing systems is their vulnerability to concept drift and out-of-distribution (OOD) anomalies. In classical MLPs, activation functions, such as ReLU or Leaky ReLU, extend indefinitely (f(x)=x for x>0), providing stable—albeit linear—extrapolation for anomalous sensor spikes.

Conversely, KANs define their univariate functions over a bounded grid (typically initialized between [−1,1] after data normalization). Physical sensors deployed in dynamic environments frequently encounter concept drift, such as a gradual increase in baseline temperature due to machine degradation or extreme vibration peaks caused by mechanical shocks. When a sensor reading falls outside the established spline grid, the KAN cannot naturally evaluate it.

To address this limitation, current implementations rely on dynamic grid extension, a process in which grid boundaries are recalculated and spline control points are updated during operation. However, this dynamic updating is computationally expensive and may become unstable during real-time edge inference. If the grid is extended too frequently due to noisy sensor spikes, the model may suffer from severe accuracy degradation. Developing KAN architectures with more robust and computationally efficient out-of-distribution extrapolation mechanisms, possibly via hybrid designs combining splines with asymptotically linear boundary functions, remains a crucial challenge for industrial and environmental sensing applications.

### 8.4. The “Wiggling” Effect and High-Frequency Sensor Noise

Physical sensor measurements are inherently noisy because of electromagnetic interference, quantization errors, and environmental artifacts. Conventional neural networks’ piecewise-linear behavior provides implicit regularization against high-frequency noise, but the high expressive capacity of KANs can become a double-edged sword in noisy sensing environments. Their practical implementation often raises issues related to numerical stability and function selection [107].

Because KANs typically employ high-order B-splines or polynomial bases (often degree k≥3), the resulting univariate functions possess sufficient flexibility to closely fit the training set’s noise distribution. Recent robustness studies further suggest that standard KAN architectures can display structural sensitivities when exposed to perturbed or noisy data [108]. This phenomenon, commonly referred to as the “wiggling” effect, occurs when the learned spline oscillates rapidly between control points to minimize local loss, resulting in severe overfitting.

KAN architectures require explicit regularization terms to mitigate overfitting and numerical instability risks. A common approach to ensure function smoothness is to penalize the second-order derivative of the univariate functions, which effectively acts as a curvature penalty:

(5) $$L_{smooth}=\lambda \sum_{e\in \mathrm{edges}}\int_{x_{min}}^{x_{max}}{\left|\frac{d^{2}\phi_{e}\left(x\right)}{dx^{2}}\right|}^{2}dx$$

where λ is the regularization coefficient. This penalty discourages high-frequency oscillations (the “wiggling” effect) in the learnable splines, ensuring that the model captures the underlying physical trends of the sensor data rather than fitting high-frequency noise.

However, tuning the hyperparameter λ across hundreds of heterogeneous sensor channels—for example, balancing smooth temperature signals with naturally oscillatory acoustic emission signals—remains a challenging optimization problem. Therefore, future work should develop modality-aware regularization strategies that suppress sensor noise without removing critical high-frequency transient patterns.

### 8.5. Hyperparameter Explosion in Multimodal Fusion

Designing a sensor-fusion MLP or CNN in conventional deep learning typically involves selecting network width, depth, and learning rate. The introduction of KAN architectures dramatically expands the hyperparameter search space, posing a significant barrier to their widespread adoption in complex IoT and cyber–physical systems.

For each KAN layer, practitioners must now select:

- Basis function type: B-splines, Chebyshev, Fourier, or Jacobi polynomials.
- Grid resolution (*G*): The number of intervals used to define the spline grid. A fine grid captures intricate sensor patterns but increases the risk of overfitting and memory consumption, whereas a coarse grid acts as a low-pass filter.
- Polynomial degree (*k*): Determining the smoothness and flexibility of the learned function.

This issue becomes particularly critical in multi-modal sensor fusion. Consider a healthcare IoMT system that integrates a high-frequency ECG sensor (500Hz) with a low-frequency body temperature sensor (0.1Hz). Applying a uniform grid size *G* across all sensor channels is suboptimal: the ECG signal requires a high-resolution grid to capture QRS complexes, whereas the temperature sensor requires a much coarser grid.

Currently, tuning these hyperparameters for each sensor modality typically relies on expensive grid-search procedures. Automated KAN architecture search (KAN-NAS) frameworks, which can dynamically assign grid resolutions and basis functions to individual sensor edges during training, represent a significant research area for future multimodal KAN deployments.

### 8.6. Edge and Embedded Deployment

Many sensor-driven applications require real-time processing on edge platforms with limited computational resources. KAN-based models, despite being parameter efficient, still need further optimization for deployment in resource-constrained environments. Therefore, future studies should focus on lightweight KAN variants. Recent work, such as the TCNN-KAN model [90], has shown the feasibility of real-time edge deployment through unstructured pruning, indicating that accelerator-aware design will be essential for practical IoMT and industrial sensing systems.

### 8.7. Standardized Benchmarks and Evaluation Protocols

The lack of standardized benchmarking is a major limitation of the current literature. Establishing common datasets and reproducible evaluation pipelines is essential [12,13]. For example, the use of the MIMIC-III and MIMIC-IV datasets in studies such as TCKAN [87] provides a useful baseline for multimodal sensor integration. Future evaluation protocols should extend beyond predictive accuracy to include metrics related to energy consumption, robustness to noise—as investigated in MSFKAN [81]—and interpretability.

### 8.8. Design Guidelines for KAN-Based Sensing Systems

Future research should aim to establish systematic guidelines for selecting basis functions for specific sensing tasks, potentially through neural architecture search (NAS) methods mentioned in [22,29]. Moreover, linking learned univariate functions to physical variables—such as the integration of temporal and constant clinical data in TCKAN—may help bridge the gap between purely data-driven models and domain knowledge.

To summarize the main open challenges and the research directions proposed to address them, Table 12 provides a structured overview of current limitations and potential future developments for KAN-based sensing systems.

## 9. Conclusions

This survey examined the rapidly growing body of work on KANs for sensor data processing, covering 58 selected studies published between 2024 and 2026. Research from the industrial, biomedical, and remote sensing sectors has demonstrated a consistent trend, where KAN-based architectures generally surpass or equal traditional deep learning models in predictive accuracy, while utilising fewer parameters and offering greater functional transparency than fixed-activation networks. The most effective deployments reviewed here do not treat KANs as standalone replacements for CNNs or Transformers. KAN modules are integrated into key locations within existing pipelines, including classification heads, bottleneck layers, and physics-constrained components, where the capacity to learn interpretable univariate functions provides a quantifiable improvement. Models such as KACNet, KANSeg, AutoKAN, and MSFKAN illustrate this hybrid strategy across a variety of sensing modalities and task types.

In this context, KAN-based architectures are likely to play a key role in the development of next-generation sensing systems, where interpretability, robustness, and computational efficiency are simultaneously required.

Despite these advantages, several obstacles remain before the large-scale deployment of KAN-based sensing systems. The computational cost of spline evaluation is substantially higher than that of equivalent matrix operations, creating a concrete bottleneck for real-time edge inference despite favorable parameter counts. The lack of consensus on basis-function selection, the sensitivity of high-order splines to sensor noise, and the absence of standardized benchmarks across sensing domains impede the reproducibility and comparability of current results.

Looking ahead, three research directions stand out as particularly critical for KAN-based sensing system maturation. Due to the heterogeneity of datasets and experimental conditions, which complicates the comparison of current results, establishing standardized benchmarks and evaluation protocols across sensing domains is a pressing priority. The adoption of shared repositories, such as MIMIC-IV for clinical sensing or curated hyperspectral datasets for remote sensing, will enable reproducible comparisons. Second, the sensitivity of high-order splines to sensor noise necessitates the development of modality-aware regularization strategies. Unlike MLPs, whose piecewise-linear activations provide an implicit low-pass effect, KANs require explicit smoothness constraints to prevent overfitting high-frequency noise artifacts. Future work must investigate adaptive schemes capable of distinguishing genuine transient anomalies from measurement noise through hybrid spline–linear formulations.

Third, bridging the gap between parameter efficiency and computational latency is essential for practical edge deployment. Because spline evaluation is currently memory bound, KANs do not fully benefit from optimized GEMM kernels. Sparse KAN topologies mapped onto FPGAs or neuromorphic processors, which are hardware-aware designs, seem to be one of the most promising directions for resource-constrained implementation. Progress in these areas will determine whether KANs have moved from being a promising research frontier to becoming a foundational component of next-generation intelligent sensing systems. Therefore, future research should focus on scalable KAN architectures, automated basis-function selection, and hardware-aware implementations capable of supporting real-time processing in large-scale sensor networks.

## Figures and Tables

**Table 1:** Positioning of this survey with respect to existing review papers on Kolmogorov–Arnold Networks ^†^.

Survey	Year	Theory	Variants	Applications	Sensor Focus	Cross-Domain
Dutta et al. [44]	2025	✓	✓	Partial	–	–
Somvanshi et al. [12]	2025	✓	✓	Partial	–	–
Ji et al. [13]	2025	✓	✓	✓	Limited	–
Yamak et al. [46]	2025	✓	✓	TSF only	Limited	–
Wang et al. [45]	2025	✓	✓	✓	Limited	Partial
This survey	2026	✓	✓	✓	✓	✓

^†^ Sensor focus refers to systematic coverage of physical sensing modalities and deployment constraints. This survey covers three domains: industrial and mechanical sensing, medical and biomedical sensing, and remote sensing and environmental monitoring. ✓ indicates comprehensive coverage; Partial indicates coverage of some domains or aspects but not all; Limited indicates brief or superficial coverage; – indicates not covered.

**Table 2:** Basis functions used for KAN edge-wise parameterization.

Family	Generic Edge Function	Definition/Properties
B-splines [10]	$\phi \left(x\right)=\sum_{m}\theta_{m}B_{m,r}\left(x\right)$	Piecewise-polynomial basis with local support. Ideal for high-frequency sensor data and “grid extension” refinement.
Chebyshev [22]	$\phi \left(x\right)=\sum_{k=0}^{K-1}\theta_{k}T_{k}\left(\tilde{x}\right)$	Favorable minimax approximation properties; minimizes maximum error over bounded sensor intervals.
Legendre [53]	$\phi \left(x\right)=\sum_{k=0}^{K-1}\theta_{k}P_{k}\left(\tilde{x}\right)$	Global orthogonal polynomials. Effective for modeling smooth global trends in long-term degradation signals.
Hermite [54]	$\phi \left(x\right)=\sum_{k=0}^{K-1}\theta_{k}H_{k}\left(x\right)$	Associated with Gaussian-weighted expansions; suitable for normalized physicochemical variables with normal distributions.
Laguerre [54]	$\phi \left(x\right)=\sum_{k=0}^{K-1}\theta_{k}L_{k}\left(x\right)$	Orthogonal on [0,∞) with weight $e^{-x}$. Optimized for non-negative magnitudes such as sensor-measured viscosity or soot concentration.
RBFs [55]	$\phi \left(x\right)=\sum_{k=1}^{K}\theta_{k}\exp\left(-\frac{{\left(x-c_{k}\right)}^{2}}{2\sigma_{k}^{2}}\right)$	Gaussian radial basis functions; effective for capturing sharp transitions and localized patterns in contamination indicators.

**Table 3:** Representative variants of Kolmogorov–Arnold Networks and their sensing applications.

Variant	Basis	Typical Use Case	Key Advantage
Spline-KAN	B-splines	General sensor fusion	High local adaptability
Chebyshev-KAN	Chebyshev	Scientific sensing	Enhanced numerical stability
Jacobi-KAN	Jacobi	Physics-informed systems	Tunable inductive bias
Conv-KAN	Splines + CNN	Hyperspectral imaging	Spatial–spectral feature learning
Graph-KAN	KAN + GNN	Wireless sensor networks	Nonlinear relational modeling
MonoKAN	Hermite Splines	Physical systems	Certified partial monotonicity for safety-critical outputs

**Table 4:** Summary of literature search results and study selection across KAN-based sensing domains (status as of March 2026) ^†^.

Sensing Domain	Scopus	Cited ≥ 1 (%)	Web of Science	Cited ≥ 1 (%)	Selected
Industrial and Mechanical	142	46.5%	202	59.9%	25
Medical and Biomedical	174	37.9%	187	44.9%	15
Remote Sensing	84	45.2%	73	56.2%	18
**Total**	**400**	**42.5%**	**462**	**60.2%**	**58**

^†^ Values in bold indicate the final aggregated results across all sensing domains, distinguishing them from individual domain entries.

**Table 5:** Consolidated summary of selected studies on KAN-based architectures in industrial and mechanical sensing.

Ref.	Domain	Architecture	Key Contribution
[73]	Soft Sensors	AKGNN	Graph optimization + KAN inference
[32]	Soft Sensors	TCN-KAN	Multi-timescale features
[74]	Ind. Process	KAGCN-KATCN	Graph + Temporal KAN
[75]	Wastewater	KAN	Bio-electrocatalytic modeling
[30]	Fault Diagnosis	KAN	Feature selection + XAI
[78]	RUL Prediction	Trans.-KAN-WP	Hybrid stochastic drift
[59]	Fault Diagnosis	CNN-1D-KAN	Cross-domain transfer
[79]	Failure Pred.	Performer-KAN	IGBT monitoring
[65]	Tool Monit.	MTF-AViTK	2D Signal + ViT-KAN
[66]	Quality Ctrl.	ISet Trans.-KAN	Feature importance
[69]	Cybersecurity	KAN	Lightweight IDS for CPS
[72]	IIoT Anomaly	TCN-VAE-KAN	Unsupervised + XAI
[71]	IIoT Offload	D2KCO (DRL)	Task offloading optimization
[70]	DDoS Det.	CNN-mLSTM-KAN	Depthwise conv. + KAN
[3]	Time-series	C-KAN	Conv. KAN + DILATE loss
[33]	Energy Syst.	KAN_PCC	PCC-based plant optimization
[63]	Welding Opt.	KAN-GA	Interpretable surrogate + SHAP
[60]	Process FDD	KAN-AE	Data-efficient detection
[61]	Spectroscopy	BSSDN + KAN	Denoising + Trace analysis
[62]	Elemental	PGNAA + KAN	γ-ray spectral mapping
[64]	Animal Health	HRDS	Lightweight HRNet + DS Att.
[67]	Device Mod.	KAN-based SR	Gray-box SPICE model generation via formulas
[76]	Aquaponics	KAN-based SR	Extraction of formulas for WQI; sensor pruning
[77]	Chem. Proc.	KAN Soft Sensor	SR for industrial interpretability
[68]	General TSF	HiKAN	Integrates IEKAN for seasonal modeling and AFM.

**Table 6:** Summary of KAN-based architectures in medical sensing.

Ref.	Domain	Architecture	Key Contribution
[8]	PET-CT/MR	RT-KAN	Real-time segmentation in hybrid imaging.
[80]	Lung Cancer	MobileNet+KAN	Ensemble classification approach for diagnosis.
[81]	Multimodal	MSFKAN	Multi-scale feature fusion for clinical data.
[82]	2D/3D Imaging	KACNet	Integration of KANConv modules in standard backbones.
[83]	Segmentation	U-KAN	KAN-based U-Net architecture for medical masks.
[84]	Multi-organ	KANSeg	Introduction of KAN-ACM and KAN-BM blocks.
[85]	UTI Diagnosis	K2AN/KAN-C-N	Visual CFU recognition for automated diagnosis.
[42]	Respiratory	KAN	Dust exposure detection in occupational health.
[86]	Imaging	DEQ-KAN	Equilibrium-based infinite-depth modeling.
[38]	EEG/Seizures	KAN vs. LSTM	Nonlinear analysis of temporal brain signals.
[39]	EEG	KAN-EEG	Enhanced geographic generalization for signal processing.
[87]	Sepsis/Clinical	TCKAN	Multimodal integration for critical care monitoring.
[88]	IoMT/Diabetes	AutoKAN	Federated anomaly detection for patient privacy.
[90]	sEMG	TCNN-KAN-2	Unstructured pruning for edge deployment.
[89]	Genomics	KAN	Identification of drug–gene associations.

**Table 7:** Summary of KAN-based architectures in remote sensing (2024–2026).

Ref.	Domain	Architecture	Key Innovation
[91]	LULC	KonvNeXt	First KAN-RS implementation with occlusion-based interpretability.
[92]	HSI Class.	Hybrid 1/2/3D	Multi-dimensional feature extraction for hyperspectral data.
[5]	HSI Class.	HyperKAN	Modular replacement of Linear, Conv, and Attention layers with KANs.
[94]	HSI Class.	HyperFKAN	Fourier-based KANs designed for high-speed spectral processing.
[95]	HSI Class.	HPFN	Hierarchical progressive fusion for lightweight classification.
[4]	HSI Class.	KAN-Transformer	Enhanced Fusion Transformer with Kolmogorov–Arnold layers.
[93]	HSI Class.	HSS-KAMNet	Spectral–spatial KAN optimization for airborne sensors.
[7]	UAV Detect.	KSCNet	Collaboration between KAN-YOLO and State Space Models (SSM).
[96]	Segment.	SS-KAN	Self-supervised depthwise KAN for limited labeled data scenarios.
[97]	Flood Monit.	FloodKAN	SAR-based flood extraction utilizing nonlinear spline activations.
[34]	Change Det.	AEKAN	Unsupervised Siamese Autoencoder for multi-temporal analysis.
[98]	Change Det.	Cheb-KAN	Chebyshev polynomial basis for signal noise reduction in change masks.
[99]	Image Fusion	SFIGNet	Spatial–frequency dual domain integration for pansharpening.
[100]	Image Fusion	KanDiff	Diffusion models with KAN-guided integration for heterogeneous sensors.
[101]	Denoising	Wavelet-KAN	Wavelet-based texture preservation in remote sensing imagery.
[37]	Agriculture	KAN	Interpretable crop yield prediction using multi-source satellite data.
[102]	Hydrology	KAN	Chlorophyll-a prediction in lakes via nonlinear spectral mapping.
[35]	Weather	SwinKAN	Radar extrapolation for dual-polarization precipitation forecasting.

**Table 8:** Integration of KAN into deep learning architectures: a critical analysis of practical trade-offs for sensor data.

Base Arch.	KAN-Integrated Models	References	Critical Analysis/Practical Trade-Offs
AE	AEKAN	[34]	Enhances nonlinear noise representation in sensor reconstruction, but increases training complexity compared to standard MLPs.
CNN	C-KAN, SwinKAN, KACNET	[3,35,82]	Improves spatial feature interpretability (activation visualization). However, evaluating splines significantly increases inference latency on resource-constrained edge devices.
Transformers	RT-DEKT, KAN-Transformer, HiKAN	[4,8,68]	Reduces parameter reliance for attention mechanisms, yet lacks standardized benchmarking to validate stability in large-scale SCADA deployments.

**Table 9:** Benchmark comparison across RGB RS datasets. All results are reported from [93].

Dataset	Model	F1	AUC
EuroSAT	HSS-KAMNet	99.39	99.62
	EfficientNet-B7	98.30	98.67
	Swin Transformer	98.61	99.01
	YOLOv8-Cls	97.68	97.90
UCM	HSS-KAMNet	98.31	98.71
	Swin Transformer	97.35	97.61
	MobileNetV3	94.41	94.88
AID	HSS-KAMNet	98.05	98.54
	EfficientNet-B7	92.47	93.12
	Swin Transformer	93.55	94.20

**Table 10:** Benchmark comparison on hyperspectral datasets (adapted from [92]).

Dataset	Model	OA (%)	AA (%)	κ
Tangdaowan	ResNet-50	98.09	96.99	97.82
	HybridKAN	98.08	97.12	97.81
Pingan	VGG-16	99.06	98.09	98.61
	HybridKAN	98.48	95.95	97.74
Qingyun	HybridKAN	97.06	94.91	96.11
	2D-CNN	96.98	95.60	96.00

**Table 11:** Cross-domain summary of KAN-based sensing architectures and their impact across the three systematically reviewed domains.

Domain	Data Modality	Key Architectures	Reported Impact
Industrial	Vibrations, SCADA, spectroscopy	KAN-AE, CNN-1D-KAN, TCN-KAN	Superior fault diagnosis XAI, data-efficient anomaly detection, and cross-domain transfer.
Medical	MRI, CT, EEG, sEMG, genomics	KACNet, KANSeg, TCKAN, AutoKAN	Accurate organ boundary segmentation, multimodal risk prediction, and federated IoMT monitoring.
Remote Sensing	HSI, SAR, optical, lidar	HyperKAN, AEKAN, WKAN-UNet, SwinKAN	Enhanced spectral–spatial mapping, flood extraction, and weather extrapolation.

**Table 12:** Summary of open challenges and proposed research directions for KANs in sensing.

Challenge	Current Limitation	Future Direction
Scalability	Memory and latency limitations in large networks.	Sparsity, distributed KAN training, adaptive grids.
Computation	Spline evaluation is slower than matrix multiplication.	Hardware-aware KAN design and kernel optimization.
Deployment	High energy cost on edge devices.	Pruning, quantization, lightweight KAN variants.
Standardization	Lack of unified datasets and evaluation metrics.	Public KAN sensing benchmarks (e.g., MIMIC-IV).
Design Theory	Heuristic basis-function selection.	NAS-based KAN architecture discovery.

## Data Availability

No new data were created or analyzed in this study. Data sharing is not applicable to this article.

## Abbreviations

The following abbreviations are used in this manuscript:

AA	Average Accuracy
ACDC	Automated Cardiac Diagnosis Challenge
AE	Autoencoder
AEKAN	AutoEncoder Kolmogorov–Arnold Network
AKGNN	Adaptive KAN-based Graph Neural Network
ANN	Artificial Neural Networks
AUC	Area Under the Curve
BiLSTM	Bidirectional Long Short-Term Memory
BSSDN	Blind-Spot Self-Denoising Network
CDWL	Coherent Doppler Wind Lidar
CFU	Colony-Forming Units
C-KAN	Convolutional KAN
CNN	Convolutional Neural Network
CPS	Cyber–Physical System
CT	Computed Tomography
DEQ-KAN	Deep equilibrium Kolmogorov–Arnold Networks
DDoS	Distributed Denial-of-Service
DRL	Deep Reinforcement Learning
DTCO	Design Technology Co-Optimization
DSC	Dice Similarity Coefficient
ECG	Electrocardiogram
EEG	Electroencephalography
FPGA	Field-Programmable Gate Array
GA	Genetic Algorithms
GDKansformer	Group-Dynamic KANSformer
GEMM	General Matrix Multiply
GNN	Graph Neural Network
GPU	Graphics Processing Unit
HPFN	Hierarchical Progressive Fusion Network
HSI	Hyperspectral Imaging
HRDS	HRNet with Dim-Channel and Space Gate Attention Using Kolmogorov–Arnold Networks
HiKAN	Hybrid Imaginary Exponential KAN
ICD	International Classification of Diseases
IDS	Intrusion Detection System
IEKAN	Imaginary Exponential basis
IGBT	Insulated-Gate Bipolar Transistor
IIoT	Industrial Internet of Things
IoMT	Internet of Medical Things
IoT	Internet of Things
ISAR	Inverse Synthetic Aperture Radar
KACNet	Kolmogorov–Arnold Convolutional Network
KAGCN-KATCN	Kolmogorov–Arnold graph convolutional aggregation temporal convolutional network
KAN	Kolmogorov–Arnold Network
KAN-ACM	KAN-Activated Convolution Module
KAN-AE	KAN-AutoEncoder
KAN-BM	KAN Bottleneck Module
KAN-NAS	Automated KAN Architecture Search
KANConv	KAN Convolutional Module
KART	Kolmogorov–Arnold Representation Theorem
LIBS	Laser-Induced Breakdown Spectroscopy
LiDAR	Light Detection and Ranging
LSTM	Long Short-Term Memory
LULC	Land-Use and Land-Cover
MLP	Multi-Layer Perceptron
MRI	Magnetic Resonance Imaging
MSI	Multispectral imaging
MSFKAN	Multi-Scale Feature Kolmogorov–Arnold Network
MTF	Markov Transition Field
NAS	Neural Architecture Search
OA	overall accuracy
OOD	Out-of-Distribution
PCC	Pearson Correlation Coefficient
PET-CT	Positron Emission Tomography–Computed Tomography
PGNAA	Prompt Gamma Neutron Activation Analysis
PINN	Physics-Informed Neural Network
PHM	Prognostics and Health Management
PRISMA	Preferred Reporting Items for Systematic Reviews and Meta-Analyses
PPG	Photoplethysmogram
QCT	Quantitative Computed Tomography
RBF	Radial Basis Function
RNN	Recurrent Neural Network
RUL	Remaining Useful Life
SAR	Synthetic Aperture Radar
SCADA	Supervisory Control and Data Acquisition
SCKansformer	Sparse-Causal KANSformer
sEMG	surface Electromyography
SFIGNet	Spatial–Frequency Sampling Implicit Neural Representation
SHAP	Shapley Additive Explanations
SiLU	Sigmoid Linear Unit
SOTA	State-of-the-Art
SPICE	Simulation Program with Integrated Circuit Emphasis
SR	Symbolic Regression
SSIM	Structural Similarity Index Measure
SSM	State Space Model
TCN	Time Convolutional Network
TCKAN	Time-Constant Kolmogorov–Arnold Network
TPU	Tensor Processing Unit
TSF	Time Series Forecasting
UAT	Universal Approximation Theorem
UAV	Unmanned Aerial Vehicle
UTI	Urinary Tract Infection
VAE	Variational AutoEncoder
ViT	Vision Transformer
WoS	Web of Science
WP	Wiener Process
WQI	Water Quality Indices
XAI	Explainable Artificial Intelligence
